# A 3D‐Printed Vascular‐Like Perfusion Tumor‐on‐a‐Chip Recapitulates High‐Grade Breast Cancer With in Vivo‐Relevant Drug Resistance

**DOI:** 10.1002/adhm.71457

**Published:** 2026-07-30

**Authors:** Geonhui Lee, Bingjie Sun, Nicholas X. Fang, Chunguang Xia

**Affiliations:** ^1^ BMF Precision Inc. Maynard Massachusetts USA; ^2^ The University of Hong Kong Hong Kong China; ^3^ Materials Innovation Institute for Life Sciences and Energy (MILES) HKU‐SIRI Shenzhen China

**Keywords:** 3D printing, drug resistance, high‐density tumor model, precision medicine, tumor‐on‐a‐chip, vascular‐like perfusion microfluidics

## Abstract

Tumor progression and drug resistance in breast cancer are fundamentally governed by a complex microenvironment, characterized by extreme cell densities and associated nutrient/oxygen gradients. Conventional in vitro models often fail to replicate these features due to mass transport limitations in non‐perfused systems. Here, we present a next‐generation tumor‐on‐a‐chip platform engineered via high‐resolution projection micro‐stereolithography (PµSL) to sustain exceptionally high‐density breast cancer tissues (up to 400 million cells/mL) under continuous perfusion. By integrating a vascular‐inspired perfusion architecture with precision‐engineered micropores (∼7 µm), our system enhances nutrient and drug transport compared with traditional non‐perfused 3D cultures. This high‐density microenvironment drives density‐dependent molecular reprogramming of ERα, Ki67, and CTSL2, closely mirroring the gene expression signatures observed in high‐grade patient tumors. Notably, the drug resistance to Elacestrant observed in our high‐density tissues uniquely aligns with clinically reported Cmax concentrations, effectively bridging the gap between in vitro screening and in vivo outcomes. This platform provides a physiologically relevant tool for studying aggressive tumor biology and evaluating personalized therapeutic strategies with high predictive power.

## Graphical abstract

A high‐resolution 3D‐printed tumor‐on‐a‐chip platform sustains breast cancer tissues at in vivo–like cell densities through vascular‐like perfusion. This engineered system recapitulates grade‐dependent molecular features and exhibits drug‐resistance profiles that uniquely align with clinically relevant Cmax values. It provides a physiologically relevant framework for investigating aggressive tumor biology and evaluating personalized therapeutic strategies with enhanced predictive potential.

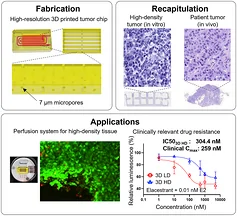

## 1 Introduction

The interplay between physiologic tissue structure and function has gained significant attention, yet the contributions of specific anatomical components to overall tissue function remain unclear [1]. Investigations into actual cell number and density in human tissues have provided critical insights into the complexities of the in vivo microenvironment [2, 3, 4]. High‐density tumor tissues, in particular, exhibit unique properties such as nutrient and oxygen gradients, hypoxia, and altered paracrine signaling, which are pivotal in driving cancer progression, metastasis, and drug resistance [5, 6]. However, conventional 2D cell cultures and basic tumor‐on‐a‐chip models fail to replicate these features, limiting their ability to accurately simulate the tumor microenvironment and predict therapeutic outcomes.

A major hurdle in constructing in vitro models of high‐density tumor tissues is achieving both the requisite cell density and microenvironmental complexity. High‐grade breast tumors, which are distinguished by densely packed cellular architectures, exhibit hallmark behaviors such as elevated drug resistance and increased metastatic potential [7, 8, 9]. Although vascularized 3D culture systems have been explored, recreating tumor densities comparable to those observed in vivo remains challenging. Without effective vascular‐like perfusion, these models cannot establish the perfusion dynamics necessary to sustain realistic tissue densities and functional gradients [10, 11, 12, 13, 14, 15].

High‐density tumor tissues are particularly critical for dissecting the dynamics of estrogen receptor‐alpha (ERα) expression and its influence on drug resistance, metastasis, and overall cancer progression [16, 17, 18, 19]. Tumor grade, a classification determined by factors such as tubule formation, nuclear pleomorphism, and mitotic count, is often correlated with tumor density, with higher‐grade tumors exhibiting denser cellular architectures. These densely packed tumors undergo distinctive gene expression shifts, including changes in ERα, a key biomarker for hormone receptor‐positive breast cancer [20, 21, 22, 23]. High‐grade tumors are notorious for their aggressive behavior, rapid growth, and heightened resistance to therapy, emphasizing the urgency of understanding their underlying mechanisms. Clinical evidence increasingly links dense breast tissue, frequently found in high‐grade tumors, to significant alterations in gene expression profiles. Notably, genes such as ERα, Ki67 (a proliferation marker), and CTSL2 (associated with tumor invasion and drug resistance) show density‐dependent regulation. These molecular signatures not only reflect tumor aggressiveness but also point to potential therapeutic targets [24]. However, without in vitro models that accurately recapitulate these high‐density conditions, our understanding of ERα regulation, as well as the broader pathways driving drug resistance and metastasis, remains limited. Filling this gap, advanced in vitro models capable of simulating high‐density tumor tissues can provide valuable insights into how tumor grade and density contribute to breast cancer progression.

In this study, we introduce a next‐generation, vascular‐like perfusion tumor‐on‐a‐chip model designed to replicate the high‐density architecture of breast cancer tissues. Leveraging a 3D‐printed vascular‐like scaffold, our platform sustains continuous perfusion, enabling the culture of exceptionally dense tumor tissues at cell densities reaching 400 million cells/mL. This capability provides a unique opportunity to investigate critical aspects of breast cancer biology, including tumor progression, ERα regulation, and drug resistance, under physiologically relevant conditions. Additionally, direct comparisons of ERα, Ki67, and CTSL2—key genes from the Oncotype DX 21‐gene test—between our in vitro high‐density tissues and patient‐derived data, reveal parallel density‐ and grade‐dependent gene expression trends. By capturing these key molecular signatures, our innovative platform not only enhances our understanding of the underlying mechanisms driving breast cancer aggressiveness but also refines drug efficacy evaluations and informs the development of personalized therapeutic strategies.

## 2 Results

### 2.1 Replicating High‐Density Tumor With a 3D‐Printed Vascular‐Like Perfusion System

**FIGURE 1:**
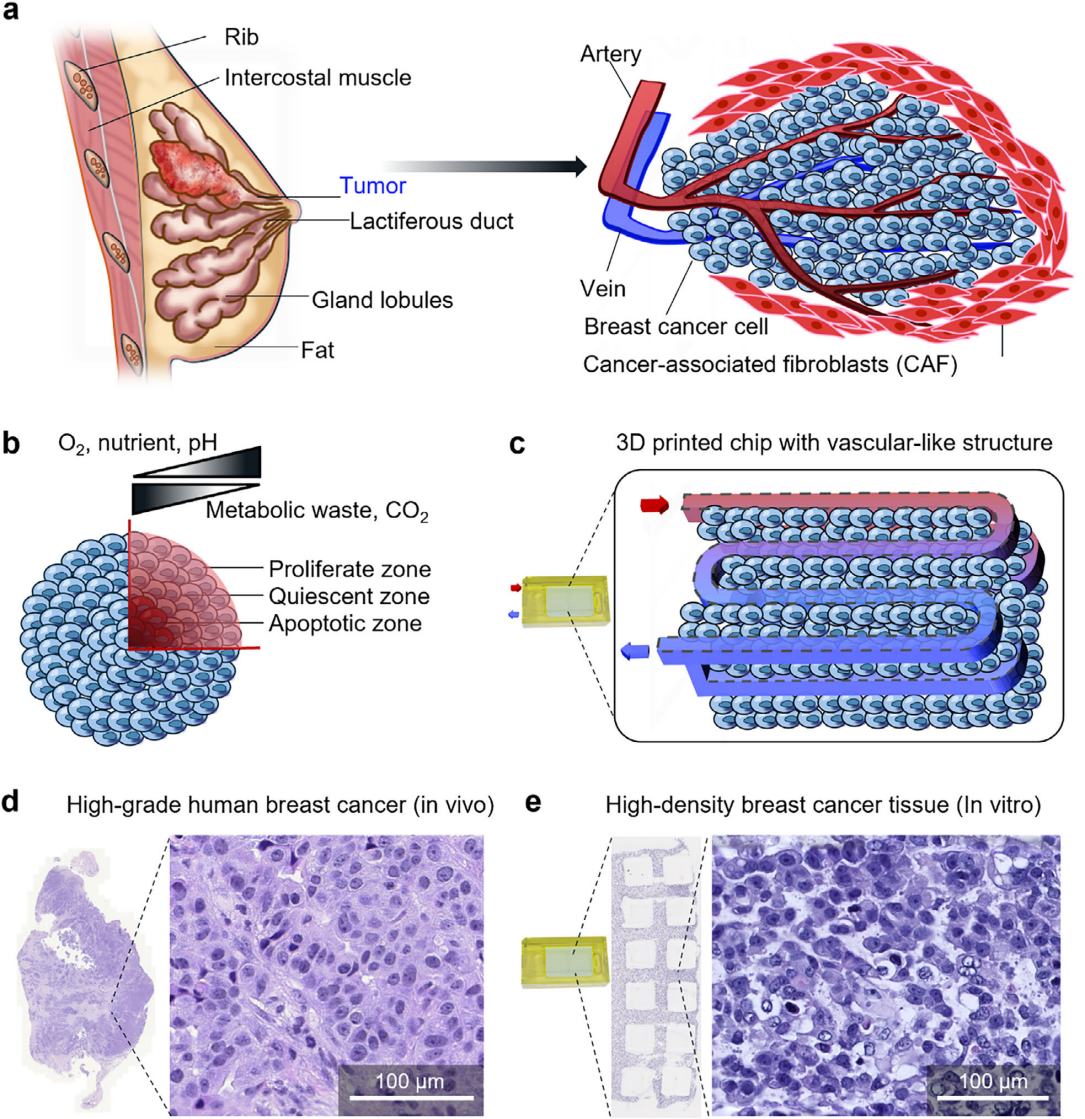
Conceptual overview of a vascular‐like perfusion breast cancer‐on‐a‐chip model. (a) Schematic illustration of high‐grade breast cancer tissue, highlighting high‐density breast cancer cells surrounded by cancer‐associated fibroblasts. (b) Cross‐sectional view of a tumor spheroid, showing three distinct zones: proliferative (outer), quiescent (middle), and apoptotic (core). (c) Schematic diagram of the 3D‐printed vascular‐like structure, featuring an inlet (red arrow), and outlet (blue arrow) to mimic artery and vein flow, respectively. (d) Representative histological image of high‐grade human breast cancer from the TCGA‐BRCA dataset. (e) Representative image of high‐density breast cancer tissue cultured in the 3D‐printed vascular‐like perfused system.

The microenvironment of high‐grade breast cancer is marked by densely packed tumor cells surrounded by cancer‐associated fibroblasts (activated HDFs) and supported by a complex vascular‐like network that supplies nutrients and oxygen (Figure 1a) [25]. Although traditional 3D spheroid models have advanced our understanding of tumor biology, they inherently lack perfusion, restricting nutrient and oxygen diffusion and preventing the formation of high cell densities comparable to those observed in vivo (Figure 1b). We utilized high‐resolution PµSL to engineer a vascular‐like microfluidic network capable of sustaining extreme cellularity (Figure 1c). Histological analysis confirmed that our in vitro high‐density tissue (Figure 1e) recapitulates the extreme cellularity and nuclear‐to‐cytoplasmic ratios characteristic of Grade 3 human breast cancer tissues (Figure 1d). This morphological alignment demonstrates that our platform can physically replicate the architectural hallmarks of aggressive malignancy.

### 2.2 Characterization of the 3D‐Printed Breast Cancer on a Chip

**FIGURE 2:**
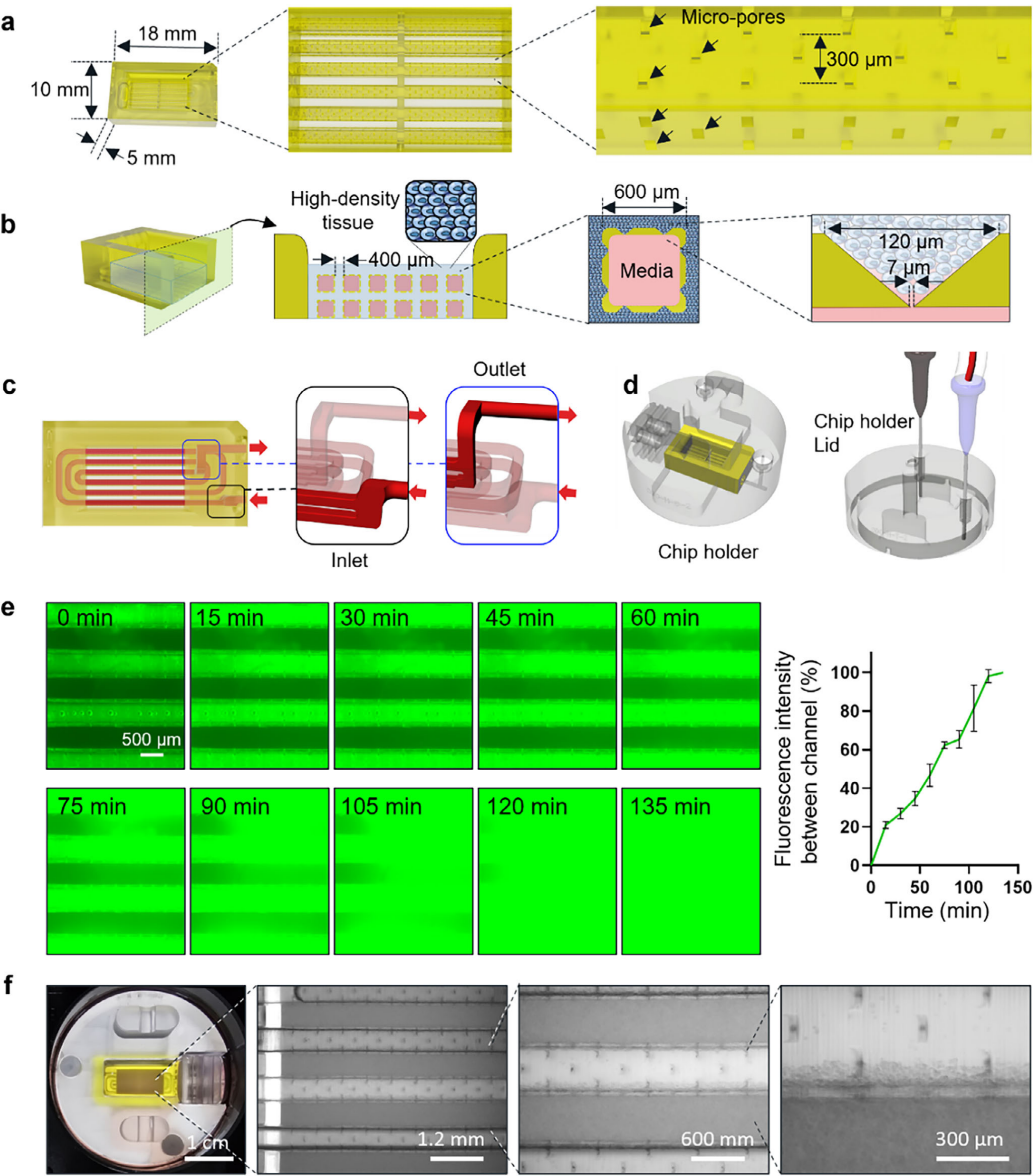
Characterization of the 3D‐printed vascular‐like perfusion breast cancer‐on‐a‐chip model and its diffusion system. (a) Design of the 3D‐printed vascular‐like perfusion system, featuring six parallel channels embedded with 7 µm micro‐pores to facilitate nutrient and oxygen exchange. (b) Schematic illustration of high‐density breast cancer tissue within the 3D‐printed vascular‐like perfusion system. (c) Flow schematic of the 3D‐printed vascular‐like perfusion system, showing fluid entry at the inlet, subsequently bifurcation into two layers, and recombination at the outlet, ensuring uniform perfusion. (d) Design of the customized chip holder and lid, compatible with standard 6‐well plate formats for streamlined handing and testing. (e) Timelapse imaging of FITC‐dextran diffusion through the micro‐pores in the chip, at a flow rate of 10 µL/min, with fluorescence intensity monitored over time. Statistical analysis was performed using an unpaired two‐tailed t‐test. Error bars represent standard deviations (SD) (*n* = 3). (f) Representative image of the breast cancer‐on‐a‐chip model, showing MCF7 cells seeded at high density within collagen I matrix, recapitulating the dense architecture characteristic of high‐grade breast cancer tumors.

The microfluidic chip was fabricated using projection micro‐stereolithography 3D printing technology with BIO‐resin from Boston Micro Fabrication (BMF), allowing the precise formation of microchannels and micropores. Each hollow microchannel in the chip features evenly spaced micropores (∼7 µm) that facilitate more efficient nutrient delivery to the tissue compared to traditional hydrogel‐based devices with slower flow rates (Figure 2a and Table S1). Furthermore, the BIO‐resin minimizes drug adsorption, unlike hydrogels or polydimethylsiloxane (PDMS), which are commonly used in organ‐on‐a‐chip systems [26, 27]. The chip measures 10 × 18 × 5 mm and is specifically engineered to support large, dense tissue cultures. It comprises two layers of vascular‐like, six‐channel structures with micro‐pores (∼7 µm) optimized for nutrient diffusion. Recognizing that the diffusion is limited to approximately 200–300 µm [28, 29], we spaced the channels 400 µm apart to ensure sufficient nutrient delivery (Figure 2b). To further enhance diffusion homogeneity and avoid blockage due to extracellular matrix (ECM) polymerization, we implemented spiral‐shaped microfluidic channels instead of traditional binary tree‐like pattern (Figure 1c). Additionally, we developed a customized polycarbonate chip holder and lid compatible with standard 6‐well plates, facilitating streamlined handling and scalability (Figure 2d). For high‐throughput experiments, we incorporated a 12‐channel peristaltic pump (6 channels for inlets and 6 for outlets) (Figure S1a,b).

To confirm uniform nutrient distribution, we conducted a diffusion test by monitoring the time‐lapse fluorescence intensity of FITC‐dextran (MW: 4000) after ECM polymerization in the chip. Quantitative image analysis revealed that fluorescence intensity reached full saturation in approximately 2 h, indicating effective transport through the micropore network under the tested condition (100 million cells/mL) (Figure 2e). To further evaluate transport behavior at higher tissue densities, we performed transport simulations using the experimental chip geometry. The 100 million cells/mL condition reproduced the experimentally observed diffusion trend, while simulations using a more conservative effective diffusion coefficient for the 400 million cells/mL condition predicted slower but sustained solute penetration throughout the tissue compartment (Figure S5). These results provide additional quantitative support that the micropore architecture remains capable of supporting transport in high‐density tissues. Lastly, to validate the platform's capability to support breast cancer tissue formation, we cultured MCF7 (ER+) breast cancer cells at a high ECM concentration (4 mg/mL collagen). The resulting tissue formed without blocking the microchannels, demonstrating our approach for maintaining a functional, high‐density tumor environment (Figures 2f and S1c–e).

### 2.3 Activated HDF Enhances Proliferation and Transmigration of Breast Cancer Cells

**FIGURE 3:**
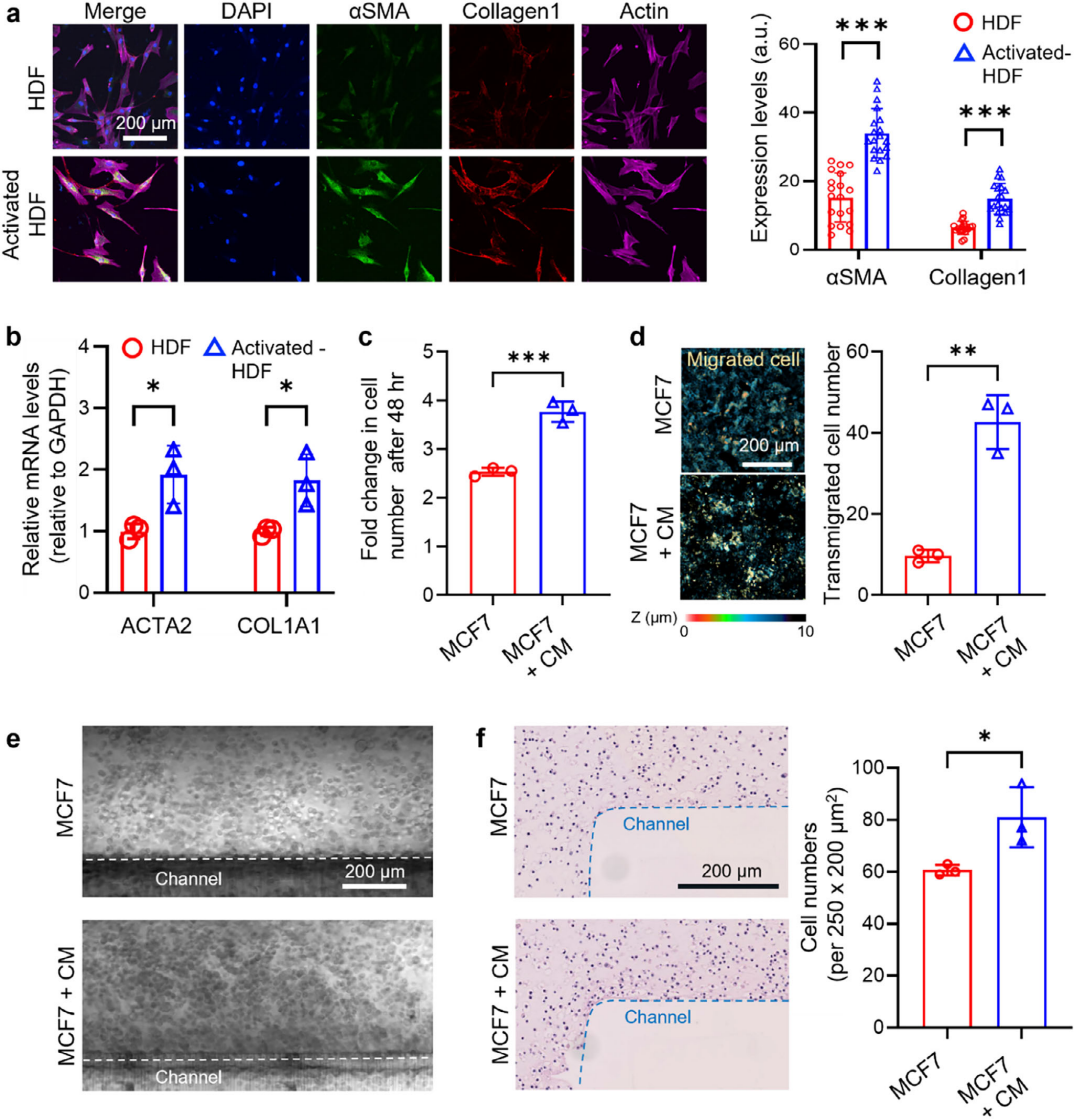
Activated HDF enhances proliferation and transmigration of breast cancer cells. a. Representative fluorescence images of HDFs and activated HDFs. HDFs were activated with 10 ng/ml TGF‐β for 48 h to induce activated HDF phenotypes (*n* = 19). (b) Comparison of mRNA expression levels of ACTA2 and COL1A1 in HDFs and activated HDFs, showing upregulation of these markers in activated HDFs. (c) Fold change in the proliferation of MCF7 cells cultured with activated HDF‐conditioned medium (CM) compared to controls. (d) Transwell invasion assay of MCF7 cells in the presence of activated HDF‐CM. Noninvasive MCF7 cells were imaged above the membrane, while invasive cells were imaged below the membrane after 48 h. Cell counts were performed in a 500 × 500 µm area. (e) Representative images of MCF7 cells cultured in the 3D‐printed vascular‐like perfusion system. (f) Representative cross‐sectional H&E‐stained images of MCF7 cells cultured in the 3D‐printed vascular‐like perfusion system. Statistical analyses were conducted using an unpaired two‐tailed t‐test. Error bars represent SD (*p < 0.05, **p < 0.01, and ***p < 0.001, ns: not significant). Scale bar: 200 µm.

Activated HDFs are known to play a critical role in breast cancer progression by enhancing cancer cell proliferation, invasion, and metastasis [30, 31, 32, 33]. To investigate this effect in our high‐density breast cancer tissue model, we activated human dermal fibroblasts (HDFs) with 10 ng/ml of TGF‐β. Activation of HDFs was confirmed by significant upregulation of CAF markers, ACTA2, and COL1A1, at both the protein and mRNA levels through fluorescence imaging (Figure 3a) and quantitative PCR (qPCR) assays (Figure 3b), compared to nonactivated HDFs. Additionally, a significant increase in cell area was observed in activated HDFs relative to HDFs suggesting morphological changes associated with activation (Figure S2a).

Next, we evaluated the effects of activated HDF‐conditioned medium (activated HDF‐CM) on the proliferation of MCF7 breast cancer cells. Fold‐change analysis revealed a significant enhancement in MCF7 cell proliferation in the presence of activated HDF‐CM compared to control conditions (Figure 3c). To further assess the activated HDF‐CM‐induced invasiveness, we performed a Transwell invasion assay. Imaging of the upper and lower chambers of the Transwell membrane showed a marked increase in transmigration of MCF7 cells in the presence of activated HDF‐CM compared to controls (Figures 3d and S2b).

Further, we investigated whether activated HDF‐CM promotes proliferation in high‐density breast cancer tissues within our chip model. In our 3D‐printed vascular‐like perfusion system, MCF7 cells treated with activated HDF‐CM exhibited significantly higher cell density. Differential Interference Contrast (DIC) and cross‐sectional Hematoxylin and Eosin (H&E) staining images revealed enhanced tissue density and structural integrity in the presence of activated HDFs (Figures 3e,f and S2c).

These findings highlight the crucial role of activated HDFs in promoting breast cancer cell progression and their importance in the tumor microenvironment. Our results validate the potential of our 3D‐printed chip system for modeling dynamic activated HDFs‐cancer cells interactions, offering a powerful tool for investigating tumor biology and advancing therapeutic strategies.

### 2.4 Optimization of Flow Rate for High‐Density Breast Cancer Tissue

To determine optimal conditions for sustaining high‐density breast cancer tissues, we compared static and flow‐based culture systems. In static cultures with varying seeding densities, cell viability decreased significantly as densities increased. Specifically, cultures seeded at 25 million cells/mL retained approximately 70% viability after 24 h, while those at 50 million cells/mL dropped to about 30%, and those at 100 million cells/mL exhibited less than 10% viability due to severe nutrient limitations (Figure S3a). These results highlighted the critical need for flow‐based systems to support the viability of dense cultures.

Flow culture experiments were performed at a seeding density of 100 million cells/mL, using flow rates of 2, 5, and 10 µL/min. Among these, a flow rate of 10 µL/min was identified as the minimum required and sufficient flow rate to maintain high cell viability (> 90%) and ensure uniform tissue distribution (Figure S3b). Quantitative image analysis confirmed substantial improvements in cell viability at this flow rate compared to the lower rates.

These findings highlight the effectiveness of our 3D‐printed microfluidic chip in generating high‐density breast cancer tissues with minimal flow requirements. Our chip achieved homogeneous and viable tissues at low flow rate, 10 µL/min, demonstrating its efficiency in delivering nutrients and oxygen to the dense cell cultures. The media is introduced via the main channels, and the flow within the tissue chamber is primarily diffusive interstitial flow. Simulations results estimated that interstitial shear stress within the tissue compartment remained below 0.1 dyne/cm^2^ under the tested perfusion condition (Figure S6), providing optimal nutrient delivery without detrimental mechanical stress.

### 2.5 Flow Enhances Viability and Proliferation in High‐Density Breast Cancer Tissue

**FIGURE 4:**
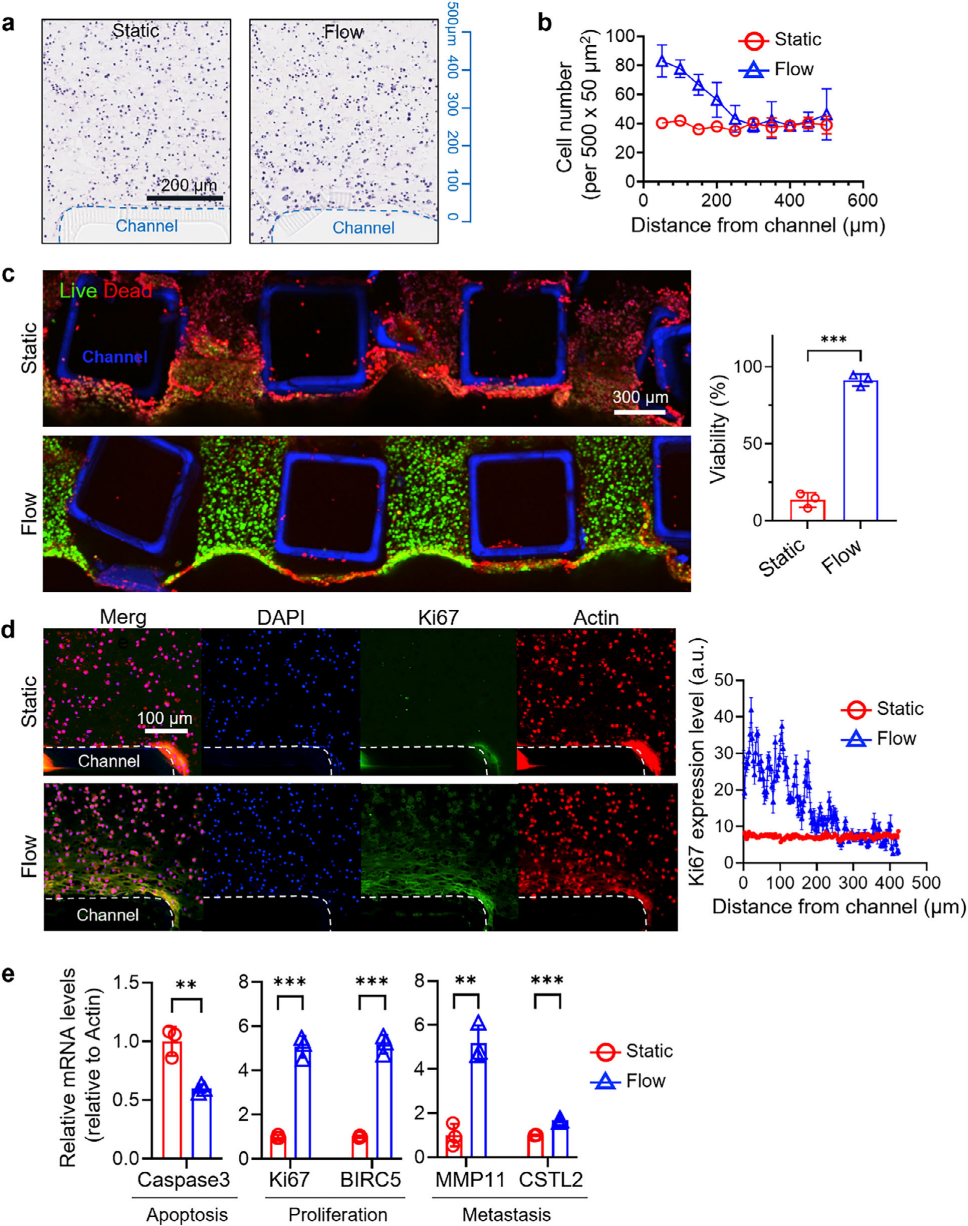
Flow enhances viability in high‐density breast cancer tissue. (a) Representative cross‐sectional H&E‐stained images of MCF7 cells under static and flow conditions. Scale bar: 200 µm. (b) Quantitative analysis of cell numbers at varying distances from the microchannel. (c) Representative live/dead staining images and viability quantification of MCF7 cells in static and flow conditions. Scale bar: 300 µm. (d) Representative fluorescence images of MCF7 cells under static and flow conditions. Scale bar: 100 µm. (e) Comparison of mRNA expression levels of apoptosis‐, proliferation‐, and metastasis‐related markers in MCF7 cells under static and flow conditions. Statistical analyses were performed using an unpaired two‐tailed t‐test. Error bars represent SD (*p < 0.05, **p < 0.01, and ***p < 0.001, ns: not significant).

We next assessed the role of flow in maintaining high‐density breast cancer tissues within our microfluidic chip. Cross‐sectional H&E staining revealed a higher concentration of cells near the microchannels, with a gradual decrease in density extending toward the top of the ECM‐filled chamber. This gradient reflects effective nutrient diffusion across the 400 µm channel spacing, emphasizing the advantage of our microfluidic design in mimicking physiological nutrient gradients (Figure 4a,b).

To quantify the effects of flow on cell viability, we performed live and dead assays. Under flow conditions, cell viability achieved approximately 90%, compared to only ∼10% viability in static cultures, highlighting the essential role of perfusion in sustaining cell health (Figure 4c). Further, we analyzed Ki67 expression, a key proliferation marker, through fluorescence imaging. The results revealed a proliferation gradient, with cells nearer to the microchannels exhibiting significantly higher proliferation, consistent with nutrient availability (Figure 4d).

Additionally, we observed reduced levels of apoptotic markers (Caspase 3) and increased expression of proliferation markers (Ki67, BIRC5) and metastatic markers (MMP11, CSTL2) in flow cultures compared to static conditions, suggesting that the dynamic flow environment nurtures a more aggressive tumor phenotype (Figure 4e).

Collectively, these findings confirm that our microfluidic platform effectively supports cell viability, promotes proliferation, and maintains spatial organization in high‐density breast cancer tissues, providing a physiologically relevant model for studying tumor dynamics.

### 2.6 High‐Density Breast Cancer Tissue Expressed Patient Gene Expression Profiles

**FIGURE 5:**
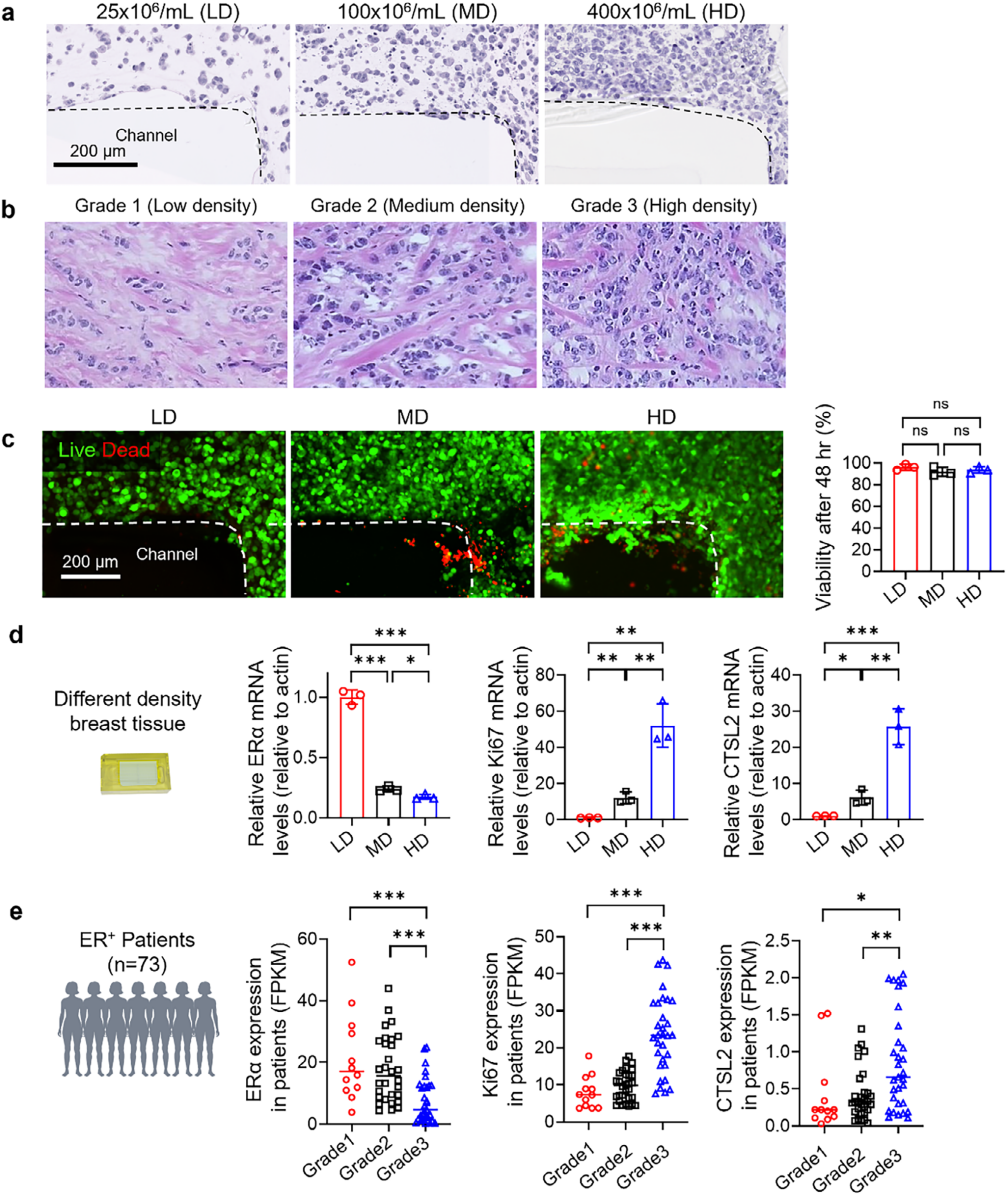
High‐density breast cancer tissues in the chip recapitulate gene expression patterns observed in ER+ patients. (a) Representative H&E‐stained images of MCF7 cells cultured at low, medium, and high densities in the chip. (b) Representative H&E‐stained images of grade 1, 2, and 3 breast cancer tissues from patients, showing histological similarities with in vitro tissues (Images captured at 20x magnification). (c) Representative live/dead staining images and viability analysis of breast cancer tissues cultured in the chip at different densities after 48 h. (d) Comparison of mRNA expression levels of ERα, Ki67, and CTSL2 in MCF7 cells across varying‐density breast cancer tissues in the chip. (e) Comparison of mRNA expression levels of ERα, Ki67, and CTSL2 in 73 ER+ breast cancer patients. Statistical analyses were performed using an unpaired two‐tailed t‐test. Error bars represent SD (*p < 0.05, **p < 0.01, and ***p < 0.001, ns: not significant). Scale bar: 200 µm.

Next, to replicate different grades of breast cancer, we adjusted cell density in our 3D chip model. The Nottingham histological grading system assigns scores from 1 to 3 based on factors such as tubular formation, nuclear pleomorphism, and mitosis [34, 35]. Higher‐grade tumors exhibit greater microvessel density and tumor cellularity [36, 37]. To model this, we cultured breast cancer tissues in our 3D chip using three different cell densities: low density (LD, 25 million cells/mL), medium density (MD, 100 million cells/mL), and high density (HD, 400 million cells/mL), which correspond to histological grades 1, 2, and 3 in breast cancer patients, respectively, (Figure 5a). H&E staining showed that the cell density of LD, MD, and HD tissues in our model closely resembled those of grades 1, 2, and 3 tumors in patient samples (Figure 5b).

We confirmed thatin HD tissues maintained high cell viability (>90%) over 48 h experimental period at a flow rate of 10 µL/min (Figure 5c). Interestingly, mRNA expression analysis of MCF7 cells demonstrated a decrease in ERα expression with increasing cell density, while levels of Ki67 and CTSL2, markers of cell proliferation and cancer progression, significantly increased (Figure 5d). These findings were further corroborated by quantitative fluorescence imaging, which also showed reduced ERα expression at higher cell densities (Figure S4). Importantly, the gene expression trends observed in our model aligned with RNA‐seq data from 73 ER+ breast cancer patients, supporting the notion that our model successfully recapitulates the patient‐specific tumor dynamics seen in different tumor grades (Figure 5e).

These findings establish that our 3D chip enables the culture of dense breast cancer tissues that replicate both structural and molecular characteristics of patient tumors, highlights its potential as a reliable platform for studying tumor biology and evaluating therapeutic strategies.

### 2.7 Drug Resistance in High Density Breast Cancer Tissue

**FIGURE 6:**
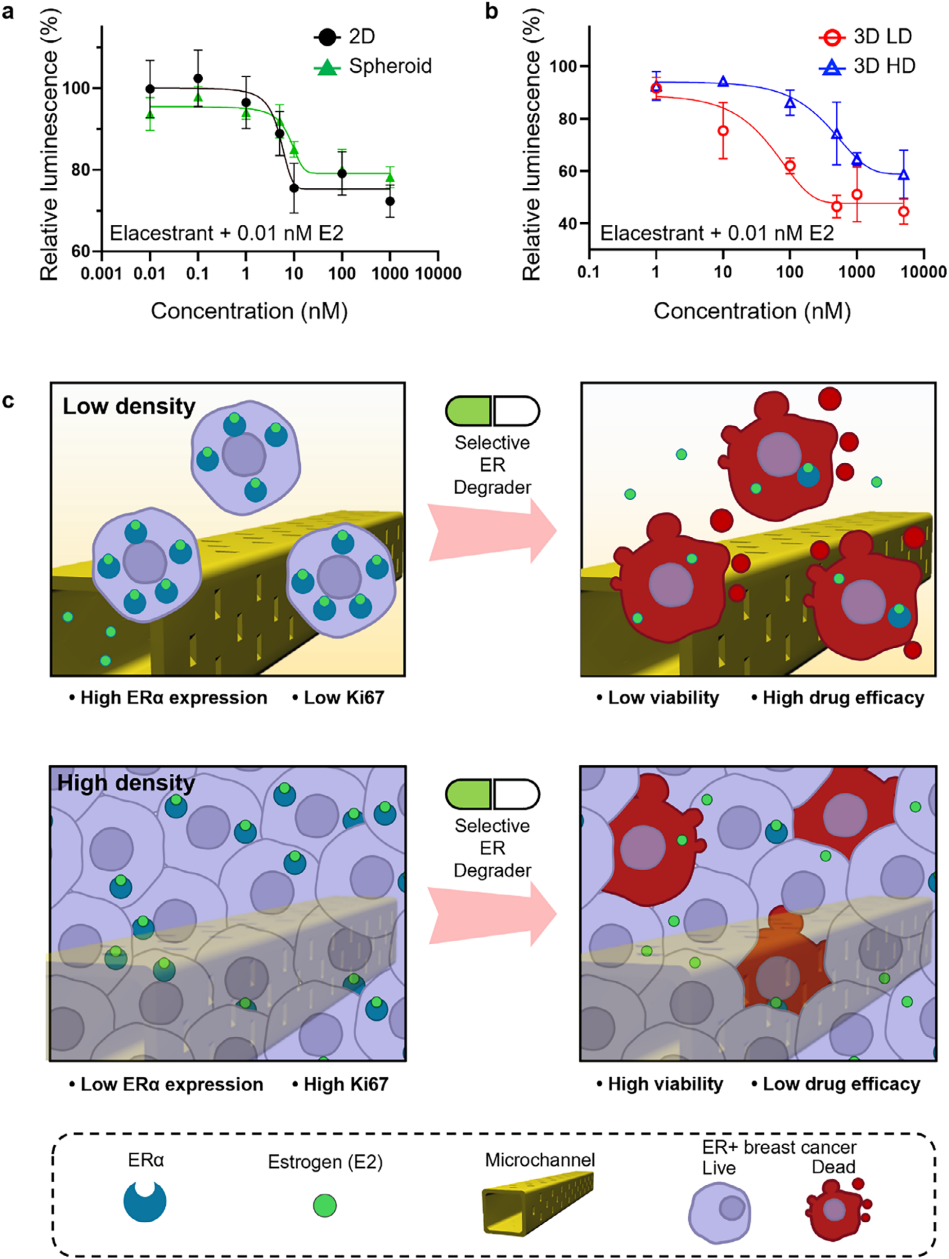
In vivo‐like high‐density breast cancer tissue exhibits enhanced drug resistance compared to spheroid and 2D cultures. (a) IC_50_ values of breast cancer cells treated with drug in 2D and spheroid models. Error bars represent SD (*n* = 3). (b) IC_50_ values of breast cancer cells treated with the same drug in 3D high‐density breast cancer tissue model, showing significantly greater resistance. Error bars represent SD (*n* = 3). (c) Schematic representation of drug responses in low‐ and high‐density breast cancer tissues, illustrating the distinct microenvironmental influences on drug penetration and efficacy.

Our results demonstrated that the high‐density breast cancer tissues cultured in our 3D chip exhibited significantly greater drug resistance compared to conventional 2D and spheroid models. Specifically, the IC_50_ for Elacestrant was 4.991 nM in 2D cultures and 7.893 nM in spheroid models. Compared with 2D cultures, spheroids exhibited a modest increase in Elacestrant resistance, likely reflecting enhanced cell‐cell interactions and transport limitations commonly associated with spheroid architecture. However, spheroid resistance remained substantially lower than that observed in the low‐density and high‐density perfused chip models. By bypassing these traditional 3D bottlenecks, our system sustains high‐density (400 M cells/mL) without the size‐restricted necrotic constraints inherent in spheroids, allowing for a more accurate evaluation of density‐dependent resistance. In contrast, low‐density tissue (25 million cells/mL) in the 3D chip had an IC_50_ of 45.215 nM, while high‐density tissue (400 million cells/mL) exhibited an even higher IC_50_ of 304.348 nM (Figure 6a,b).

Notably, the observed IC50 alignment with reported clinical exposure levels suggests improved physiological relevance of the high‐density model (Figure S6). While theoretical diffusion maps provide surrogate estimates, this clinical‐empirical correlation at the highest density tier demonstrates that our precision‐engineered funnel‐shaped micropores featuring a 120 µm entrance and a 7 µm constriction micropore network facilitates sufficient interstitial transport to reach the dense tissue core. To support the technical integrity of our scaffold, we previously confirmed rapid saturation via FITC‐dextran diffusion assays at a density of 100 million cells/mL (Figure 2e). This alignment highlights the unique ability of our chip to replicate the drug resistance patterns seen in dense in vivo tumors [38]. By capturing these in vivo‐like resistance mechanisms, our 3D chip model provides a physiologically relevant platform for preclinical drug testing. Unlike conventional models, it accounts for the influence of tumor density and microenvironmental complexity on therapeutic response. These findings highlight the advantages of our platform in evaluating drug efficacy under conditions that better mimic the challenges of treating aggressive, high‐grade tumors in clinical settings. Furthermore, the 3D chip offers valuable insights into the mechanisms underlying drug resistance, paving the way for the development of more effective personalized therapies (Figure 6c).

## 3 Discussion and Conclusion

Understanding the balance between essential and nonessential anatomical components in tissue engineering, such as cell density and functional architecture, remains a critical challenge. While certain elements may be vital for achieving specific biochemical or physiological outputs, others could simply represent evolutionary redundancies or structural byproducts. This distinction is particularly crucial when designing synthetic tissues and functional models, particularly in high‐density cellular microenvironments. While clinical tumor grading is multifactorial, we isolated cell density as an independent biophysical variable. This approach is supported by clinical studies showing a strong correlation between high mammographic/cellular density and aggressive tumor biomarkers [39, 40, 41]. Here, we leveraged advanced 3D printing techniques to address these challenges, creating a vascular‐like perfusion tumor‐on‐a‐chip model capable of sustaining high‐density breast cancer tissues during the experimental culture period. Accordingly, the term “vascular‐like” is used to describe the overall perfusion architecture rather than a physiological endothelialized microvascular model. Our findings reveal the importance of precise spatial arrangement and optimized microenvironmental conditions for achieving native‐like functionalities. This aligns with the observations of Niklason and Langer, who highlighted the critical role of cell density in regulating morphogenesis and tissue homeostasis [42].

The density‐dependent regulation of ERα, Ki67, and CTSL2 observed in our model highlights the profound impact of the physical microenvironment on tumor molecular profiles. The observed downregulation of ERα in high‐density conditions suggests that the aggressive phenotypes and therapeutic resistance seen in high‐grade clinical tumors are significantly influenced by cellularity and associated gradients. Crucially, the observed translocation of ERα from the cytoplasm to the plasma membrane under high‐density conditions (Figure S4) indicates a shift toward non‐genomic signaling pathways. This membrane‐associated ERα activity is a known adaptation to the high mechanical pressure and metabolic stress characteristic of the high‐grade tumor core, providing a rapid survival advantage and a potent mechanism for drug resistance. Such phenotypic reprogramming—which is entirely absent in sub‐physiological 2D or low‐density 3D cultures–‐likely explains why our high‐density model achieved an IC50 for Elacestrant (304.3 nM) that parallelizes the clinical Cmax (259 nM), whereas conventional models do not. This alignment suggests that conventional models fail to capture the biophysical‐to‐molecular signaling shifts that drive resistance in vivo.

Furthermore, our study provides insights into the paracrine role of the stroma. By utilizing activated HDF‐CM, we isolated the specific influence of soluble factors, demonstrating that they enhance both proliferation and structural integrity in dense tissue contexts. The utilization of established cell lines (MCF7) for initial validation was a strategic choice to minimize biological noise and ensure technical reproducibility. This standardized approach allowed us to rigorously attribute observed phenotypic shifts—such as ERα translocation and drug resistance—directly to the engineered microenvironment. Establishing this high‐fidelity baseline is a scientific necessity before transitioning to more heterogeneous clinical samples.

While our model ensures control and reproducibility, several challenges remain regarding scalability and adaptability to diverse organ systems. The necessity of complex vascular networks, as seen in organs like the lungs and liver, versus simplified organ‐on‐chip designs, is still under investigation. High‐precision, automated 3D printing technologies show promise in addressing these parameters. However, achieving functional integration in vivo—bridging engineered tissues with native systems—will likely necessitate further advancements in materials science and organ‐on‐a‐chip device design.

We acknowledge that direct experimental validation of solute transport at 400 million cells/mL, such as FITC‐dextran diffusion assays performed at this density, would further strengthen the transport‐related conclusions of this study. The COMSOL‐based simulations presented here provide quantitative support but are subject to the assumptions made regarding the effective diffusion coefficient at high cell density. Future studies should include direct transport measurements across the full range of tissue densities used in drug efficacy assays.

In conclusion, our 3D‐printed vascular‐like perfusion tumor‐on‐a‐chip model represents a transformative step forward in developing in vitro systems that emulate the complex characteristics of high‐density tumors in vivo. By integrating activated HDFs, optimizing perfusion, and effectively modeling drug resistance, this platform provides a robust framework for studying tumor biology, evaluating drug efficacy, and designing personalized therapeutic strategies. Notably, we validated the relevance of our model by comparing its gene expression with a dataset of 73 patient's RNA‐sequencing data, demonstrating similar results by using ERα, Ki67, CTSL2, representative genes of oncotype DX 21‐gene test. This comparison emphasizes the model's ability to accurately replicate critical aspects of the tumor microenvironment, lending strong support to its potential as a tool for preclinical evaluation of cancer treatments. Furthermore, it provides a novel avenue for exploring cancer progression and advancing precision oncology research.

## 4 Materials and Methods

### 4.1 Chip Design and Fabrication

The chip was designed using SolidWorks (Dassault Systèmes) and fabricated with a BMF microArch S230 projection micro‐stereolithography (PµSL) printer (optical resolution: 2 µm). BIO‐resin (BMF), a rigid biocompatible material meeting ISO 10993 standards, was utilized for fabrication. The scaffold architecture was strategically optimized for high‐density tissue perfusion: microchannels were spaced at 400 µm intervals to ensure that every cell within the 400 million cells/mL tissue remains within a 200 µm proximity to a perfusion source, thereby circumventing the metabolic diffusion limits (∼200 µm) and preventing necrotic core formation. Additionally, the 7 µm micropores were engineered to serve as a biomimetic selective barrier, functionally resembling capillary fenestrations; this dimension effectively retains MCF7 cells (average diameter ∼15–20 µm) within the tissue chamber while facilitating efficient interstitial fluid transport and nutrient/drug exchange. This specific pore size also ensures the structural integrity of the high‐resolution PµSL‐printed scaffold. After printing, chips were thoroughly cleaned with 70% isopropyl alcohol and UV‐cured for 30 min to ensure proper hardening.

### 4.2 Cell Culture

Human breast cancer cell lines, MCF7 and MCF7‐GFP, were purchased from ATCC and GenTarget Inc. Cells were cultured in MEM medium (ThermoFisher) supplemented with 2 mM L‐glutamine, 0.1 mM nonessential amino acids, 1 mM sodium pyruvate, 10 µg/mL bovine insulin, 10% FBS (Fetal bovine serum), and 1% PenStrep (ThermoFisher, USA) at 37°C in a 5% CO_2_ atmosphere. Primary human neonatal dermal fibroblasts (HDF, Gibco) were maintained in Human Fibroblast Expansion Basal Medium (Gibco) supplemented with low serum growth supplement (LSGS, Gibco, USA) at 37°C in a 5% CO_2_ environment. To activate HDFs into activated HDFs, cells were treated with 10 ng/mL of TGF‐β (FisherScientific) for 48 h. (activated HDF‐CM) was then collected from activated HDFs after 3 days of incubation.

### 4.3 Preparation of Chip

Before cell seeding, the chip was sterilized by immersion in 90% ethanol for 30 min, followed by a 2 h UV treatment to ensure complete sterilization. The chip holder was designed using SolidWorks (Dassault Systèmes) and fabricated by machining polycarbonate. Once sterilized, the chip was fitted with an O‐ring at the inlet and placed into the bottom half of the chip holder, which was designed to fit into a 6‐well plate. The lid of the chip holder was then positioned on top to enclose the chip, securing it in place. Tubing connected the chip holder to a peristaltic pump for continuous fluid perfusion.

### 4.4 Preparation of ECM and Cell Seeding Into Chip

Breast cancer tissue is characterized by significantly higher stiffness compared to healthy breast tissue [43]. To mimic this characteristic, breast cancer cells were cultured in a stiff ECM [44]. ECM was prepared as previously described [14, 45]. In this formulation, Collagen I serve as the primary structural scaffold at a final concentration of 4 mg/mL to recapitulate the mechanical stiffness of high‐grade tumors, while Matrigel is integrated to provide essential basement membrane components (e.g., laminin, collagen IV) for enhanced biochemical biomimicry. This was optimized to match the mechanical stiffness (1–4 kPa) characteristic of high‐grade, desmoplastic breast cancer [46]. To prepare a 1 mL ECM volume, the following components were mixed on ice to prevent premature gelation: 38.95 µL of 10× DPBS (ThermoFisher) containing calcium and magnesium, 9.7 µL of 1N NaOH, 389.5 µL of Collagen I (10.27 mg/mL, Corning), 40 µL of Matrigel (Corning), and 511.8 µL of MEM medium containing the cell suspension. The pH was adjusted to 7.4 using NaOH, and to prevent premature gelation, all components and pipette tips were kept on ice until use.

To prevent cell escape, the 6‐well plate was coated with 20 µL of Matrigel, and the chip holder with the chip was placed inside the plate. The cell‐ECM mixture was then seeded into the chip. Cell density was used at 100 million cells/mL seeding density into the chip unless it is specified. Following seeding, the chip was incubated at 37°C in a 5% CO_2_ atmosphere for 30 min to allow the ECM to crosslink and solidify. The chip holder lid was secured in place, and the inlet and outlet ports of the chip holder were connected to the peristaltic pump. The flow rate for the total device inlet flow rate was maintained at 10 µL/min prior to bifurcation into the two channel layers. The outlet withdrawal rate was set at 15 µL/min to prevent media accumulation due to pumping speed error and maintain stable operation. When culture medium volume is within the normal operating range, the outlet pump aspirates air rather than liquid, thereby preventing excess media withdrawal and maintaining stable perfusion conditions.

### 4.5 Perfusion Assay

To perform the perfusion assay, a silicone O‐ring was placed on the inlet groove of the chip, which was then fitted into the chip holder and placed inside a 6‐well plate. The chip holder lid was securely placed on top of the chip holder, ensuring that it fit into the designated grooves. Tubing and blunt needles were connected to the inlet and outlet holes on the lid, and these were connected to their respective syringes. After the ECM polymerization within the chip, FITC‐dextran (MW: 4000, Sigma‐Aldrich) was injected into the system using a syringe and syringe pump, with a flow rate set at 10 µL/min to enable proper perfusion and diffusion.

### 4.6 Immunofluorescence Microscopy

Immunofluorescence images were obtained as previously described [47, 48]. Briefly, cells cultured on a glass bottom dish were fixed with paraformaldehyde (PFA) (ThermoFisher) for 10 min at room temperature. Following fixation, the cells were permeabilized with 0.1% (*w*/*v*) Triton X‐100 (ThermoFisher) in PBS for 10 min at room temperature, then washed with PBS containing 0.1% (*w*/*v*) BSA (ThermoFisher). The cells were blocked with 5% (*w*/*v*) BSA for 30 min to prevent nonspecific binding. Primary antibodies (αSMA, Collagen1, and ERα from Cell Signaling Technology and Ki67 from R&D system) were then applied for 1 h at room temperature. To visualize cell nuclei and actin filaments, cells were stained with NucBlue (Thermo Fisher) and DyLight 650 phalloidin (Cell Signaling Technology), respectively. After washing with PBS containing 0.1% (*w*/*v*) BSA, immunofluorescence images were captured using a Nikon AX‐R confocal microscope (Nikon) with a Plan‐Apo 10× or 40× water immersion lens.

### 4.7 Cell Proliferation and Viability Assay

For the cell proliferation assay, cells were seeded at a density of 1 × 10^5^ cells per well onto a 12‐well plate and cultured for 48 h. After the incubation period, the plate was gently washed, and cells were trypsinized and collected for cell counting using a CytoSMART system (Corning). To assess cell viability following Elacestrant treatment, a CellTiter‐Glo 3D assay (Promega) was performed. The luminescence readings were compared between control and treated groups to evaluate the effects of Elacestrant on cell viability.

### 4.8 RNA Extraction and qPCR Analysis

To quantify mRNA levels, cells were collected, and RNA was extracted using the RNeasy Kit (Qiagen) following the manufacturer's instructions. Subsequently, quantitative PCR (qPCR) was performed using iTaq‐SYBR Green (Bio‐Rad) and primers from Integrated DNA Technologies. The reactions were carried out on a QuantStudio 7 Pro Real‐Time PCR System (Thermo Fisher). The following primers were used:

Ki67 (FWD: CGTTTGTTTCCCCAGTGTCT / REV: CTCCCTGCCCCTTTCTATTC), COL1A1 (FWD: GTGCTAAAGGTGCCAATGGT / REV: ACCAGGTTCACCGCTGTTAC), GAPDH (FWD: TCATGGGTGTGAACCATGAGAA / REV: GGCATGGACTGTGGTCATGAG), BIRC5 (FWD: CCACTGAGAACGAGCCAGACTT / REV: GTATTACAGGCGTAAGCCACCG), MMP11 (FWD: CCATGTAATATCTAGATAAGGTCGGA / REV: GAGTCAAGGTCGGGTGCGTGGGAAG), β‐actin (FWD: CCAAGGCCAACCGCGAGAAGATGAC / REV: AGGGTACATGGTGGTGCCGCCAGAC), CTSL2 (FWD: TCGCGTCCTCAAGGCAATC / REV: CACAGTTGCGACTGCTTTCAT), caspase3 (FWD: GGAAGCGAATCAATGGACTCTGG/ REV: GCATCGACATCTGTACCAGACC). ACTA2 (FWD: CTATGCCTCTGGACGCACAACT / REV: CAGATCCAGACGCATGATGGCA), and ERα (FWD: CCACTATGGTGTGGCATCCTGT / REV: GGTGATCTCACACTCGTTGGAG). GAPDH and β‐actin were used as normalization controls for fibroblast and breast cancer cells, respectively.

### 4.9 Histology

Breast cancer tissue constructs were fixed in 4% PFA for 1 h at room temperature, followed by two PBS washes. Post‐fixation, the tissues were carefully removed from the chip using a laser blade. The constructs were embedded in paraffin and sectioned using a microtome. For H&E staining, the sections were deparaffinized with xylene and gradually rehydrated through an ethanol series. Rehydrated sections were submerged sequentially in Harris Hematoxylin solution, acid alcohol, a bluing reagent, and Eosin‐Y solution. Stained samples were then dehydrated through graded alcohols and washed in xylene.

For immunofluorescence staining, antigen retrieval was performed on unstained sections by immersing them in a trypsin antigen retrieval solution (Abcam‐) for 30 min before proceeding with the staining protocol.

### 4.10 Analysis of Clinical Data

The TCGA‐BRCA histological dataset (Sample ID: TCGA‐A8‐A09R‐01Z) was utilized to represent high‐density breast cancer from a patient. Validation of findings was conducted using clinical RNA‐sequencing data from the GSE163882 database, which contains 222 breast cancer samples. Within this dataset, 105 ER+ breast cancer cases were analyzed. For gene expression validation, the middle 70% of data values (73 patients) in this subset were used to ensure robust analysis.

### 4.11 Computational Transport and Flow Simulation

Computational simulations were performed using COMSOL Multiphysics (COMSOL Inc., Burlington, MA, USA) to evaluate solute transport and fluid flow within the perfusion chip. The simulation geometry was generated based on the experimental chip dimensions, including the perfusion channel, micropore structures, and tissue compartment.

For transport analysis, the Transport of Diluted Species module was used to simulate FITC‐dextran diffusion. The experimentally tested 100 million cells/mL condition was modeled using an effective diffusion coefficient of 1 × 10^−10^ m^2^/s, which reproduced the experimentally observed diffusion saturation profile. To evaluate transport under high‐density tissue conditions (400 million cells/mL), an additional simulation was performed using an effective diffusion coefficient of 2.5 × 10^−11^ m^2^/s, which was conservatively selected to represent the increased transport resistance expected from substantially higher cellular packing and ECM‐associated obstruction. This modeling assumption was guided by previous studies reporting reduced macromolecular transport in dense collagen‐ and ECM‐containing tissues [49, 50]. The inlet concentration was fixed at a normalized concentration of 1, and all external boundaries were defined as no‐flux conditions except for the outlet boundary.

To estimate flow behavior within the device, laminar flow simulations were performed using the experimentally applied inlet flow rate of 10 µL/min. Culture medium was modeled as an incompressible Newtonian fluid with a density of 1000 kg/m^3^ and a dynamic viscosity of 0.001 Pa·s. Velocity magnitude and shear‐rate distributions were analyzed along the channel–micropore–tissue path. Shear stress was estimated from the simulated shear rate according to

τ=μγ

where *µ* is the medium viscosity and *γ̇* is the shear rate.

### 4.12 Drug Response Analysis

For 2D culture, MCF7 cells were pretreated with 0.01 nM exogenous estradiol (E2) (Cayman) for 48 h and then plated in a 96‐well plate at a density of 10,000 cells per well in 100 µL of culture medium. After cell attachment and full spreading, Elacestrant (0.01, 0.1, 1, 5, 10, 100, and 1000 nM, MedChemExpress) was applied for an additional 2 days. DMSO was used as a vehicle and Elacestrant was treated as 1:1000 ratio in the medium.

For spheroid culture, MCF7 cells were plated at a density of 30,000 cells per well in a 96‐well round‐bottom ultra‐low attachment microplate (100 µL/well, Corning). Cells were supplemented with 0.01 nM E2 and cultured for 2 days to allow spheroid formation. After the initial 2 days of culture, Elacestrant was applied at concentrations of 0.01, 0.1, 1, 5, 10, 100, and 1000 nM, and the cultures were maintained for an additional 2 days.

For 3D‐printed chip experiments with LD and HD cultures, cells were pre‐mixed with ECM and 0.01 nM E2 before seeding into the chip. The cultures were maintained for 2 days under perfusion with 0.01 nM E2 in the medium prior to the application of Elacestrant (1, 10, 100, 500, 1000, and 5000 nM in the medium), which was administered for an additional 2 days. The reservoir volume of peristaltic pump was 60 mL for perfusion experiment.

Following the treatments, cell viability and drug response were analyzed using the Cell Titer‐Glo 3D assay (Promega‐) according to the manufacturer's protocol. Briefly, breast tissue was collected using a laser blade (Figure S1d) and mechanically disrupted into smaller fragments with a spatula and pipette for uniform processing. Cell Titer‐Glo was then added, followed by shaking at room temperature for 5 min and incubation for 25 min at room temperature. Finally, absorbance was measured using a plate reader (Molecular Devices). The IC_50_ values for both control and treated groups were calculated and statistically compared to evaluate the effectiveness of the treatment.

### 4.13 Statistical Analysis

All experiments were performed in triplicate unless otherwise stated. All results are expressed as mean ± standard deviation (SD). Statistical significance was determined using one‐way ANOVA followed by Tukey's post‐hoc test for multiple comparisons. Significance was marked on plots by *, ** and *** for *p* < 0.05, *p* < 0.01, and *p* < 0.001 correspondingly. Statistical analyzes were performed using GraphPad Prism (GraphPad Software, USA).

## Funding

This work was funded by BMF Precision Inc.

## Conflicts of Interest

All authors are affiliated with BMF Precision, Inc., a start‐up company involved in the development of 3D printing and organ‐on‐a‐chip technologies.

## Supporting information




**Supporting File**: adhm71457‐sup‐0001‐SuppMat.docx.

## Data Availability

The data that support the findings of this study are available on request from the corresponding author. The data are not publicly available due to privacy or ethical restrictions.
