# Supplementary material for: A 3D‐Printed Vascular‐Like Perfusion Tumor‐on‐a‐Chip Recapitulates High‐Grade Breast Cancer With in Vivo‐Relevant Drug Resistance
Source: Adv Healthc Mater. 2026 Jul 30;15(31):e71457. doi: 10.1002/adhm.71457 (PMC13495843; doi:10.1002/adhm.71457)
Supplement: Supplementary file 1 [file ADHM-15-0-s001.docx]

**Supplementary Figures and table**

| Parameter | Value |
| --- | --- |
| Chip dimensions | 10×18×5 mm |
| Fluidic Layers | 2 |
| Channels per layer | 6 |
| Channel spacing | 400 µm |
| Channel width | 600 µm |
| Micropore entrance width | 120 µm |
| Micropore throat size | 7 µm |
| Flow rate (inlet) | 10 µL/min |
| Flow rate (outlet) | 15 µL/min |

**Supplementary Table. 1. Geometry summary of vascular-like perfusion tumor-on-a-chip model.**

**Supplementary Fig. 1. Schematic of the diffusion system setup and formation of in vitro breast cancer tissue.**
**a**. Schematic representation of the diffusion system, showcasing the integration of a 3D-printed vascular-like perfusion system with a peristaltic pump and media reservoir to sustain continuous perfusion. **b**. Photograph of the breast cancer-on-a-chip model during culture. **c**. Breast cancer cells seeded in the chip with a high-concentration collagen matrix. **d**. In vitro breast cancer tissue retrieved from the chip post-experiment, highlighting the intact structure. **e**. Sliced breast cancer tissue prepared for downstream assays. Scale bar: 2 mm.

**Supplementary Fig. 2. Effects of activated HDF-CM and flow on tumor progression.**
**a**. Quantitative analysis of cell area differences between HDFs and activated HDFs. **b**. Schematic representation of the invasion assay using GFP-labeled MCF7 cells. Yellow arrows indicate invasive cells migrating through the membrane. Scale bar: 200 μm. **c**. Representative cross-sectional H&E-stained images of MCF7 cells under static and flow conditions. Statistical analyses were performed using an unpaired two-tailed t-test. Error bars represent SD (***p < 0.001). Scale bar: 100 μm

**Supplementary Fig. 3. Optimization of flow rate for maintaining viability in high-density breast cancer tissues.**
**a**. Representative live/dead staining images and viability analysis of breast cancer cells under static culture conditions at various seeding densities. Cells were imaged and assessed after 24 hours. **b**. Representative live/dead staining images and viability analysis of breast cancer cells under flow culture conditions with a seeding density of 100 million cells/mL. Statistical analyses were performed using an unpaired two-tailed t-test. Error bars represent SD (ns: not significant; *p < 0.05; **p < 0.01; ***p < 0.001). Scale bar: 200 μm.

**Supplementary Fig. 4. Density-dependent decrease in ERα expression in in vitro breast cancer tissues.**
Representative fluorescence images and analysis of ERα expression in breast cancer tissues at different densities (n =31). Statistical analyses were performed using an unpaired two-tailed t-test. Error bars represent SD (***p < 0.001). Scale bar: 100 μm.

**Supplementary Fig. 5. Computational analysis of solute transport in the perfusion chip**

**a**. Simulation geometry used for transport analysis. **b**. Simulated FITC-dextran transport under the 100 million cells/mL condition. **c**. Simulated transport under the 400 million cells/mL condition using a reduced effective diffusion coefficient to account for increased tissue density.

**Supplementary Fig. 6. Computational analysis of flow behavior within the perfusion chip.**

**a**. Velocity magnitude distribution under the experimental flow condition (10 µL/min). **b**. Velocity profile along the channel–micropore–tissue path. **c**. Shear-rate profile used to estimate local shear stress within the device.
